# Denial of prescription pain medication among people who use drugs in Vancouver, Canada

**DOI:** 10.1186/s12954-024-00956-5

**Published:** 2024-03-28

**Authors:** Evelyne Marie Piret, M.-J. Milloy, Pauline Voon, JinCheol Choi, Kora DeBeck, Kanna Hayashi, Thomas Kerr

**Affiliations:** 1 British Columbia Centre On Substance Use, 1045 Howe Street, Vancouver, BC V6Z 2A9 Canada; 2 School of Population and Public Health, University of British Columbia, 5804 Fairview Avenue, Vancouver, BC V6T 1Z3 Canada; 3 Division of Social Medicine, Department of Medicine, University of British Columbia, St. Paul’s Hospital, 608-1081 Burrard Street, Vancouver, BC V6Z 1Y6 Canada; 4 School of Public Policy, Simon Fraser University, 515 West Hastings St, Vancouver, BC V6B 5K3 Canada; 5 Faculty of Health Sciences, Simon Fraser University, 8888 University Drive, Burnaby, BC V5A 1S6 Canada

**Keywords:** Pain, Pain management, Prescription denial, People who use drugs, Prescribing guidelines

## Abstract

**Background:**

People who use drugs experience pain at two to three times the rate of the general population and yet continue to face substantial barriers to accessing appropriate and adequate treatment for pain. In light of the overdose crisis and revised opioid prescribing guidelines, we sought to identify factors associated with being denied pain medication and longitudinally investigate denial rates among people who use drugs.

**Methods:**

We used multivariable generalized estimating equations analyses to investigate factors associated with being denied pain medication among people who use drugs reporting pain in three prospective cohort studies in Vancouver, Canada. Analyses were restricted to study periods in which participants requested a prescription for pain from a healthcare provider. Descriptive statistics detail denial rates and actions taken by participants after being denied.

**Results:**

Among 1168 participants who requested a prescription for pain between December 2012 and March 2020, the median age was 47 years and 63.0% were male. Among 4,179 six-month observation periods, 907 (21.7%) included a report of being denied requested pain medication. In multivariable analyses, age was negatively associated with prescription denial (adjusted odds ratio [AOR] = 0.98, 95% confidence interval [CI]:0.97–0.99), while self-managing pain (AOR = 2.48, 95%CI:2.04–3.00), experiencing a non-fatal overdose (AOR = 1.51, 95%CI:1.22–1.88), engagement in opioid agonist therapy (AOR = 1.32, 95%CI:1.09–1.61), and daily use of heroin or other unregulated opioids (AOR = 1.32, 95%CI:1.05–1.66) were positively associated with being denied. Common actions taken (*n* = 895) after denial were accessing the unregulated drug supply (53.5%), doing nothing (30.6%), and going to a different doctor/emergency room (6.1%). The period following the introduction of new prescribing guidelines was not associated with a change in denial rates.

**Conclusions:**

A substantial proportion of people who use drugs continue to be denied prescriptions for pain, with such denial associated with important substance use-related harms, including non-fatal overdose. Guidelines specific to the pharmaceutical management of pain among people who use drugs are needed.

## Background

Pain is a leading cause of years lived with disability worldwide and a major driver of healthcare engagement in North America [1–3]. People who use drugs (PWUD) experience disproportionately high rates of pain, with an estimated 48 to 60% of people who use prescription opioids non-medically reporting chronic pain compared to 15 to 21% of the general Canadian population [4–6]. In addition to the negative impacts of chronic and acute pain on health, function, and quality of life [5, 7], PWUD experience other unique consequences of pain. Recent evidence from cohorts of Medicaid recipients in the United States suggests that both chronic and acute pain are negatively associated with substance use treatment initiation and retention following a substance use disorder diagnosis or a non-fatal overdose [8, 9]. The risks associated with self-management of pain via the unregulated drug supply and increased tolerance to opioids are especially concerning given the ongoing overdose crisis across the United States and Canada [7, 10, 11]. In the Canadian province of British Columbia (BC), over 11,000 suspected illicit drug toxicity deaths have been recorded since a public health emergency was declared in April 2016, with 2293 lives lost in 2022 alone, driven by illicitly manufactured fentanyl, fentanyl analogues, and other contaminants in the unregulated drug supply [12, 13].

Canadians have been among the highest consumers of medical opioids over the last decade [14]. However, recent guidelines have sought to reduce the prescribing of medical opioids in light of the ongoing overdose crisis, the limited evidence supporting opioids as an appropriate treatment for chronic non-cancer pain compared to available alternatives, and the serious risks associated with long-term opioid use [11, 15–17]. In June 2016, the College of Physicians and Surgeons of British Columbia released new guidelines and enforceable standards to curtail the prescribing of opioids, sedatives, and stimulants [18]. Endorsing the United States’ Centers for Disease Control and Prevention’s 2016 Guideline for Prescribing Opioids for Chronic Pain, the document recommended against prescribing long-term opioid treatments to patients with substance use disorders and established stricter standards around the dosing of opioids [18–20]. Canadian federal guidelines for the prescription of opioids were released in June 2017 with comparable recommendations [21]. Early assessments of the provincial policy change found that opioid prescribing in BC continued to decline moderately following the policy’s introduction, as had been the trend prior to the policy’s implementation [22, 23]. However, there is evidence that the Canadian national guidelines are not effectively translating into practice with studies highlighting limitations regarding physicians’ understanding of, and adherence to the opioid prescribing guidelines [24, 25].

Though legitimate concerns exist around the risks of escalating or relapsing substance use, diversion, lessening efficacy, and hyperalgesia when prescribing opioids for long-term pain management [17], alternative licit therapies to manage pain are not accessible to many PWUD [26]. Existing literature highlights how PWUD suffering from acute and chronic pain experience unique and overlapping barriers to accessing care, including stigma, distrust, and discrimination within healthcare settings [26, 27]. Medication requests are often labelled as drug-seeking and illegitimate, leading to the undertreatment of pain and deterioration of patient-provider relationships [27–33]. These outcomes may be exacerbated for people who experience intersecting marginalization, including Indigenous peoples and other racialized people who face institutional and interpersonal racism [34–37]. In addition to the challenges in accessing pharmacological therapies, alternative pain management strategies such as psychotherapeutic care and physical therapy remain unattainable to many people living with persistent pain due to affordability, accessibility, and availability barriers [38]. Indeed, Dassieu, Kabore et al. (2020) describes how the daily challenges faced by PWUD experiencing numerous comorbidities and socio-economic marginalization can relegate pain and pain management to the periphery [39]. The negative consequences of unmanaged pain on socio-economic conditions and substance use among PWUD reinforce many of these barriers, creating a cycle of harm that limits opportunities for care [28, 39].

In 2010, the International Pain Summit declared that access to pain management is a fundamental human right [40]. Despite experiencing disproportionate rates of pain, PWUD face critical barriers to licit pain management, negatively affecting health and wellbeing. We sought to assess access to pharmaceutical pain therapies among structurally marginalized PWUD amidst reforms to opioid prescribing guidelines in the context of the ongoing public health emergency and heightened attention around opioid prescribing. We longitudinally examine the factors associated with PWUD being denied a prescription for pain medication and explore the actions taken after being denied. Additionally, we investigate whether the toxic drug supply and evolving policy landscape have affected the rates of denial for requested prescription pain medication by PWUD over time.

## Methods

### Study design

Data for this study were drawn from three open, ongoing, and harmonized prospective cohort studies of PWUD in Vancouver, Canada: the Vancouver Injection Drug Users Study (VIDUS), the AIDS Care Cohort to evaluate Exposure to Survival Services (ACCESS), and the At-Risk Youth Study (ARYS). These cohorts have previously been described in detail [41–43]. Briefly, these cohorts have been recruiting participants since 2005 through community-based methods including street outreach, word of mouth, and self-referral. Recruitment and follow-up activities for VIDUS and ACCESS largely focus on Vancouver’s Downtown Eastside, an urban neighbourhood with high levels of substance use, criminalization, and marginalization, while ARYS operates in the Downtown South, a similar neighbourhood with a substantial population of street-involved youth. VIDUS includes adults at risk of human immunodeficiency virus (HIV) who injected drugs in the month prior to enrolment, and ACCESS includes people living with HIV who used unregulated drugs (other than or in addition to cannabis) in the month prior to enrolment. ARYS includes street-involved youth aged 14 to 26 at risk of HIV who used unregulated drugs in the month prior to enrolment. VIDUS and ARYS participants who seroconvert to HIV-positive status during follow-up are transferred to the ACCESS cohort. All eligible participants provided written informed consent at enrolment.

At baseline and every six months thereafter, participants are invited to complete interviewer-administered questionnaires that cover a range of topics including socio-demographic characteristics, substance use practices, social-structural exposures, sexual behaviours, and harm reduction and addiction care utilization. Nurse-administered questionnaires on health status and services use are also conducted at each visit. Nurses collect urine samples for drug screening and blood samples for HIV and hepatitis C virus (HCV) serology testing or monitoring. Participants receive a $40 (CAD) honorarium at each study visit. All three cohorts have received annual approval from the University of British Columbia/Providence Health Care Research Ethics Board.

### Study sample

The present analysis was restricted to study visits occurring between December 1, 2012 and March 17, 2020, when all in-person research activities were suspended because of the COVID-19 pandemic. We included all study periods in which a participant reported pain or discomfort and having requested a prescription for pain medication in the previous six months. Beginning in June 2014, the inclusion criteria were revised to include individuals who had requested or continued a prescription for pain medication in the previous six months. Pain was assessed using the EuroQol EQ-5D-3L instrument, which asks respondents to indicate whether they have no, moderate, or extreme pain or discomfort on the day of the interview [44]. This standardized measure has been validated among people with chronic pain and PWUD [45–47].

### Study variables

The primary outcome of interest was self-reporting being denied a request for prescription pain medication during the previous six months, collected as a binary variable (yes vs. no). All potential explanatory variables considered were selected based on previous research on pain among PWUD and our extensive experience in the study setting [48, 49]. Sociodemographic characteristics included: sex assigned at birth (male vs. female); age (continuous, per year older); ethnicity/ancestry (Indigenous vs. person of colour [POC]/other vs. White); and education level (≥ vs. < completed high school). Other variables considered at each six-month study visit included: living with HIV (serological testing; yes vs. no); HCV status (serological testing; seropositive vs. seronegative); physical disability that limits mobility (yes vs. no); housing status (defined as living in a single room occupancy hotel, shelter, transitional housing, or the street versus in an apartment or house; unstable vs. stable); Downtown Eastside residency (yes vs. no); recently incarcerated (including detention, prison, or jail; yes vs. no); engaged in opioid agonist therapy (yes vs. no); non-fatal overdose (yes vs. no); and self-management of pain (defined as having managed their pain on their own; yes vs. no). Substance use variables referring to patterns of use in the previous six months included: heavy alcohol use (defined according to the United States’ National Institute on Alcohol Abuse and Alcoholism [50] criteria as averaging > 4 drinks/day or > 14/week for males and > 3 drinks/day or > 7drinks/week for females; yes vs. no); daily cannabis use (yes vs. no); daily use of any stimulant (including cocaine, crystal methamphetamine, and crack; yes vs. no); daily use of heroin or other unregulated opioid (yes vs. no); any injection drug use (yes vs. no); and any daily non-medical prescription opioid use (yes vs. no). Finally, a variable assessing a potential period effect was included to evaluate whether a change in reported denial rates for prescription pain medication occurred. The period variable was divided as before (2012 to 2015; reference level) versus after (2016 to 2020) given BC’s major increase in overdose deaths in 2016, the declaration of a public health emergency in April 2016, and the changes to BC prescribing guidelines in June 2016.

### Statistical analysis

Self-reported pain intensity among the sample was explored using descriptive statistics. Baseline characteristics, stratified by prescription pain medication denial in the last six months, were assessed using Mann–Whitney test for continuous variable and Pearson’s Chi-square test for categorical and binary variables. The proportion of participants that reported being denied a requested prescription was calculated for each six-month study interview period. Since participants could provide new observations every six months, there may be some correlation in participants denied across time periods, though reports of medication requests and denials are unique to each follow-up period. As of June 2013, follow-up visits asked about the type of prescription requested and a separate denial rate was calculated for requests that included an opioid, requests for drugs that did not include an opioid, and requests for non-specified drugs. A bivariate generalized estimating equations (GEE) with logit link function was used to test for differences between the groups’ denial rates. Analyses of factors potentially associated with prescription pain medication denial included serial measures from participants, with observations from the same person likely to be correlated. To account for within-subject correlations, we used GEE with logit link function and an exchangeable correlation structure. Therefore, data from every participant follow-up observation that met the inclusion criteria were considered in the analysis. We conducted bivariate GEE analyses to determine factors associated with being denied a requested prescription pain medication and a multivariable model using GEE was fit with all explanatory variables that reached a significance level of 0.10 in the bivariate analyses. In subanalyses, we used descriptive statistics to characterize the types of pain medication participants requested, ways participants self-managed pain, and responses to the question “what did you do after you were refused?” among those denied medications. Responses were manually categorized by the first author. All analyses were performed using R (Version 4.2.2, R Foundation for Statistical Computing, Vienna, Austria). All p-values are two-sided and considered significant at *p* < 0.05 unless otherwise stated.

## Results

From December 2012 to March 2020, among 2446 participants interviewed, 1168 (47.8%) participants reported having pain and having requested a prescription for pain medication in at least one six-month study period. The median number of included visits per participant was 2 (interquartile range [IQR]: 1–5), with a total of 4,179 study interviews included in the analysis. The median age at baseline was 47 years (IQR: 37–54), 736 (63.0%) were male, 658 (56.3%) identified as White and 450 (38.5%) as Indigenous. Of all observations included, 3,206 (76.7%) involved a report of moderate pain/discomfort and the balance (*n* = 973, 23.3%) involved a report of extreme pain/discomfort.

In total, 569 participants (48.7%) reported ever being denied pain medication during the study period. Of the 3,847 observations that specified the type of prescription requested, a majority included a request for opioids (*n* = 2534, 65.9%), followed by non-opioids (*n* = 925, 24.0%), and non-specified medications (*n* = 388, 10.1%). Multiple types of pain medication were often requested within one observation. Of note, approximately one third (*n* = 752) of the requests for opioids were seeking Tylenol 3, i.e., codeine, caffeine and acetaminophen tablets. Most of the requests for non-opioid medications included over-the-counter medications (*n* = 628) and gabapentin (*n* = 534), with muscle relaxants (*n* = 99) and cannabinoids (*n* = 49) present in less than 4% of all requests. Of the 3,032 observations that reported self-managing pain, a majority (*n* = 1824, 60.2%) characterized it as involving the use of unregulated drugs or diverted pharmaceutical medications (i.e., excluding licit pharmaceutical medications, cannabis, over-the-counter medications, and alcohol/ethanol). Table 1 presents the baseline characteristics of the sample, stratified by having been denied a prescription at study baseline, i.e., the first included observation for each participant. Here, being denied a requested prescription for pain medication was negatively associated with age and living with HIV, and positively associated with unstable housing, incarceration, opioid agonist therapy, non-fatal overdose, self-management of pain, daily stimulant use, daily heroin/unregulated opioid use, any injection drug use, and daily non-medical prescription opioid use.

**Table 1:** Baseline characteristics stratified by pain medication denial among people who use drugs in Vancouver, Canada

Characteristic	Total, *n* = 1168 (100%)	Denied medication, *n* = 294 (25.2%)	Not denied medication, *n* = 874 (74.8%)	*p –* value
Age (median, IQR)	47.0 (36.6–53.7)	43.7 (32.4–51.7)	47.8 (38.3–54.5)	< 0.001
*Sex*				
Male	736 (63.0)	194 (66.0)	542 (62.0)	0.25
Female	432 (37.0)	100 (34.0)	332 (38.0)	
*Ethnicity/Ancestry*				
Indigenous	450 (38.5)	104 (35.4)	346 (39.6)	0.42
POC/other	49 (4.2)	12 (4.1)	37 (4.2)	
White	658 (56.3)	175 (59.5)	483 (55.3)	
*Education completed*				
≥ High school	610 (52.2)	160 (54.4)	450 (51.5)	0.312
< High school	537 (46.0)	126 (42.9)	411 (47.0)	
*Living with HIV*				
Yes	459 (39.3)	96 (32.65)	363 (41.5)	0.009
No	709 (60.7)	198 (67.35)	511 (58.5)	
*HCV status*				
Seropositive	901 (77.1)	226 (76.9)	675 (77.2)	1
Seronegative	265 (22.7)	67 (22.8)	198 (22.7)	
*Physical disability*				
Yes	792 (67.8)	201 (68.4)	591 (67.6)	0.811
No	375 (32.1)	92 (31.3)	283 (32.4)	
Housing status*				
Unstable	731 (62.6)	206 (70.1)	525 (60.1)	0.002
Stable	426 (36.5)	85 (28.9)	341 (39.0)	
Downtown Eastside residency*				
Yes	646 (55.3)	170 (57.8)	476 (54.5)	0.35
No	522 (44.7)	124 (42.2)	398 (45.5)	
Incarceration*				
Yes	88 (7.5)	43 (14.6)	45 (5.2)	< 0.001
No	1078 (92.3)	250 (85.0)	828 (94.7)	
Opioid agonist therapy*				
Yes	653 (55.9)	181 (61.6)	472 (54.0)	0.028
No	515 (44.1)	113 (38.4)	402 (46.0)	
Non-fatal overdose*				
Yes	128 (11.0)	50 (17.0)	78 (8.9)	< 0.001
No	1040 (89.0)	244 (83.0)	796 (91.1)	
Self-manage pain*				
Yes	830 (71.1)	264 (89.8)	566 (64.8)	< 0.001
No	337 (28.9)	30 (10.2)	307 (35.1)	
Heavy alcohol use*				
Yes	166 (14.2)	44 (15.0)	122 (14.0)	0.746
No	1001 (85.7)	250 (85.0)	751 (85.9)	
Daily cannabis use*				
Yes	320 (27.4)	81 (27.6)	239 (27.4)	0.998
No	845 (72.4)	212 (72.1)	633 (72.4)	
Daily stimulant use*,^*†*^				
Yes	298 (25.5)	95 (32.3)	203 (23.2)	0.002
No	866 (74.1)	197 (67.0)	669 (76.5)	
Daily heroin/unregulated opioid use*				
Yes	204 (17.5)	79 (26.9)	125 (14.3)	< 0.001
No	960 (82.2)	213 (72.5)	747 (85.5)	
Any injection drug use*				
Yes	707 (60.5)	210 (71.4)	497 (56.9)	< 0.001
No	460 (39.4)	83 (28.2)	377 (43.1)	
Daily non-medical prescription opioid use*				
Yes	64 (5.5)	27 (9.2)	37 (4.2)	0.002
No	1102 (94.4)	266 (90.5)	836 (95.7)	

*P*-values were calculated by a simple logistic regression for the continuous variable age, and by normal approximation and Chi-square test for categorical and binary variables, respectively

*IQR* interquartile range, *POC* person of colour, *HIV* human immunodeficiency virus, *HCV* hepatitis C virus

^*^in the six months prior to the interview date

^†^Defined as daily cocaine, crack, or meth use

**Fig. 1:**
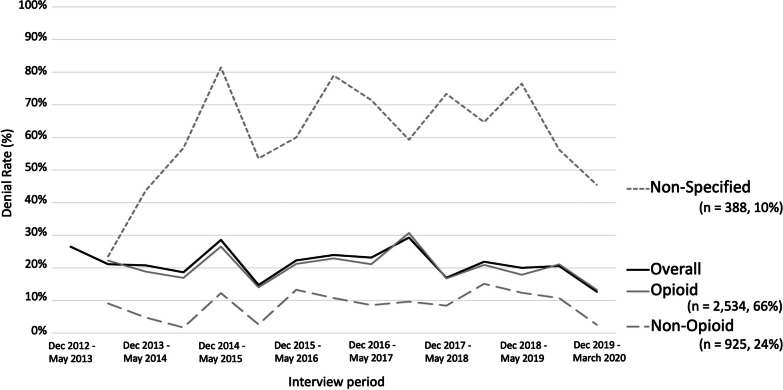
Prescription denial rates, overall and stratified by analgesic type, Vancouver, Canada, December 2012–March 2020. Prescription denial rates among 4179 participant interviews reporting pain and denial of prescription for analgesics by six-month reporting period, Vancouver, Canada, December 2012 to March 2020. * Participants were asked what pain medication they requested in June 2013 onwards, meaning these data are not available for the first interview period (n = 332). † Non-specified refers to participants who did not specify what pain medication they requested at the time of the interview (e.g., ‘anything for my back pain,’ ‘something stronger than over-the-counter medication’)

Figure 1 depicts the proportion of requests for prescription pain medications that were denied at each interview period. The overall denial rate is provided alongside differentiated denial rates for requests that included an opioid, requests that did not include an opioid, and requests that did not specify at the time of data collection (i.e., “anything for the pain”). In total, 21.7% of observations involved a denial of a request for pain medication, with the overall denial rates ranging from 12.7 to 29.3%. With respect to denial rates and ranges differentiated by type of medications requested, we observe that opioid requests had a 20.6% denial rate (range: 13.2–30.7%), non-opioid requests had a 9.3% denial rate (1.7–27.2%), and non-specified medication requests had the highest denial rate at 54.9% (23.5–81.5%). Observations with non-specified requests were significantly (*p* < 0.001) more likely to report being denied pain medication compared to observation where requests were specified (i.e., requested opioids or non-opioids).

Table 2 presents the results of the bivariate and multivariable GEE analyses, investigating factors associated with being denied a requested prescription. As shown, in bivariate GEE analyses, factors significantly and positively associated with prescription denial included: unstable housing (odds ratio [OR] = 1.26, 95% confidence interval [CI]: 1.06–1.49), incarceration (OR = 2.00, 95%CI: 1.44–2.76), engaging in opioid agonist therapy (OR = 1.38, 95%CI: 1.15–1.66), experiencing a non-fatal overdose (OR = 1.86, 95%CI: 1.52–2.28), self-managing pain (OR = 2.74, 95%CI: 2.30–3.26), daily stimulant use (OR = 1.39, 95%CI: 1.16–1.66), daily heroin/unregulated opioid use (OR = 1.91, 95%CI: 1.55–2.35), any injection drug use (OR = 1.61, 95%CI: 1.36–1.92), and daily non-medical prescription opioid use (OR = 1.36, 95%CI: 1.02–1.82). Older age (OR = 0.97, 95%CI: 0.96–0.98) and living with HIV (OR = 0.64, 95%CI: 0.53–0.79) were negatively associated with being denied a request for pain medication. The variable assessing a potential period effect was non-significant in bivariate GEE analyses, suggesting that the period after the emergency declaration and implementation of new prescribing guidelines in 2016 was not associated with a change in denial rates.

**Table 2:** Factors longitudinally associated with pain medication denial among people who use drugs in Vancouver, Canada

	Unadjusted (*n* = 4179)		Adjusted (*n* = 4134**)	
Characteristic	Odds Ratio (95% CI)	*p –* value	Odds Ratio (95% CI)	*p –* value
*Age*				
Per year older	0.97 (0.96–0.98)	< 0.001	0.98 (0.97–0.99))	< 0.001
*Sex*				
Male vs. female	1.06 (0.85–1.30)	0.616		
*Ethnicity/Ancestry*				
Indigenous vs. White	0.92 (0.75–1.13)	0.439		
POC/other vs. White	0.71 (0.41–1.25)	0.237		
*Education completed*				
≥ High school vs < High school	1.08 (0.88–1.33)	0.442		
*Living with HIV*				
Yes vs. no	0.64 (0.53–0.79)	< 0.001	0.83 (0.67–1.03)	0.088
*HCV status*				
Seropositive vs. seronegative	0.86 (0.67–1.10)	0.223		
*Physical disability*				
Yes vs. no	1.07 (0.89–1.28)	0.474		
Housing status*				
Unstable vs. stable	1.26 (1.06–1.49)	0.008	1.11 (0.92–1.33)	0.274
Downtown Eastside residency*				
Yes vs. no	1.12 (0.94–1.35)	0.208		
Incarceration*				
Yes vs. no	2.00 (1.44–2.76)	< 0.001	1.39 (0.98–1.96)	0.066
Opioid agonist therapy*				
Yes vs. no	1.38 (1.15–1.66)	0.001	1.32 (1.09–1.61)	0.005
Non-fatal overdose*				
Yes vs. no	1.86 (1.52–2.28)	< 0.001	1.51 (1.22–1.88)	< 0.001
Self-manage pain*				
Yes vs. no	2.74 (2.30–3.26)	< 0.001	2.48 (2.04–3.00)	< 0.001
Heavy alcohol use*				
Yes vs. no	1.15 (0.93–1.43)	0.191		
Daily cannabis use*				
Yes vs. no	1.16 (0.97–1.40)	0.11		
Daily stimulant use*,^*†*^				
Yes vs. no	1.39 (1.16–1.66)	< 0.001	1.12 (0.92–1.36)	0.251
Daily heroin/unregulated opioid use*				
Yes vs. no	1.91 (1.55–2.35)	< 0.001	1.32 (1.05–1.66)	0.018
Any injection drug use*				
Yes vs. no	1.61 (1.36–1.92)	< 0.001	1.12 (0.91–1.38)	0.302
Daily non-medical prescription opioid use*				
Yes vs. no	1.36 (1.02–1.82)	0.034	1.24 (0.91–1.69)	0.177
*Period*				
2016–2020 vs. 2012–2015	1.05 (0.90–1.23)	0.509		

*GEE* generalized estimating equations, *CI* confidence interval, *POC* person of colour, *HIV* human immunodeficiency virus, *HCV* hepatitis C virus

^*^in the six months prior to the interview date

^†^Defined as daily cocaine, crack, or meth use

^**^45 observations were removed from the final model due to missing data

In multivariable GEE analyses, factors that remained independently and significantly associated with being denied pain medication included: age (adjusted odds ratio [AOR] = 0.98, 95%CI: 0.97–0.99), self-managing pain (AOR = 2.48, 95%CI: 2.04–3.00), experiencing a non-fatal overdose (AOR = 1.51, 95%CI: 1.22–1.88), engaging in opioid agonist therapy (AOR = 1.32, 95%CI: 1.09–1.61), and daily heroin/unregulated opioid use (AOR = 1.32, 95%CI: 1.05–1.66).

Of the 908 participant observations that were denied a requested prescription, 895 (98.6%) reported on actions taken after being denied medication. Participants could provide more than one answer. The most common actions were accessing the unregulated drug market (*n* = 479, 53.5%), doing nothing (*n* = 274, 30.6%), and going to a different doctor/specialist/emergency room (*n* = 55, 6.1%). All other categories were reported by less than 4% of observations, and included using over-the-counter medication, alcohol or ethanol, cannabis, or someone else’s medications.

## Discussion

In our longitudinal investigation, almost half (48.7%) of the PWUD in the analytic sample reported being denied prescription pain medication at least once during the seven-year study period. Participants who were denied a prescription for pain medication were more likely to be younger and engaged in opioid agonist therapy (OAT), as well as more likely to report high-risk substance use-related characteristics, including the self-management of pain, experiencing a non-fatal overdose, and daily heroin/unregulated opioid use. Following the denial of pain medications, a majority of participants reported accessing the unregulated drug supply, a third reported doing nothing, and 6% turned to a different healthcare provider. Overall, no period effect was observed for the denial rate of requested prescriptions pain medications following changes to prescribing guidelines and the declaration of the overdose crisis, with the proportion of requests denied from 2012 to 2020 fluctuating between 13 and 29%.

Two previous studies investigating prescription pain medication denial among PWUD reported cross-sectional denial rates that fell within the range we observed, with 22.7% (34/150) and 29.2% (7/24) of participants reporting being denied [52, 53]. A third study previously conducted with the ACCESS and VIDUS cohorts prior to the current overdose crisis reported a denial rate of 66.5% (307/462) [48]. This notably higher denial rate is likely due to differences in methodological approaches and eligibility criteria, including the sample having been restricted to people engaged in active injection drug use. Although existing studies among the general population are limited and do not detail substance use patterns, one study from a family medicine clinic in California, United States reported a 18.1% (49/271) denial rate for prescription pain medication [54]. Future research is needed to further characterize denial rates among the general population, with consideration of key variables such as the type of medication requested and participants’ ability to access alternative non-pharmaceutical therapies.

In our study, people denied prescription pain medication were 2.5 times more likely to report self-managing pain, which a majority described as unregulated or diverted substance use. Self-management of pain via the unregulated drug supply is a common practice among PWUD experiencing pain [55, 56], and was reported as a direct consequence of denial by 53% of participants denied in our study. Fibbi, Silva et al. (2012) reported that among the 34 youth denied opioids for pain in their study, 18 (52.9%) reported self-managing their pain with non-medical prescription opioids or heroin [52]. Kaboré, Dassieu et al. (2020) reported that 32.1% (60/187) of their sample used non-prescription substances for pain management, though this increased to 71.4% (5/7) when restricted to those denied a prescription [53]. Though substance use is by definition common to all PWUD, there is evidence that pain increases the intensity of substance use, such as increased rates of daily substance use and injection drug use [56]. Our analysis suggests that this pattern may be heightened for PWUD who are denied pain medication, with such denial being associated with increased likelihood of daily heroin/unregulated opioid use and non-fatal overdose. Qualitative investigations of PWUD’s self-management of pain with substances reveal the complexity of the practice, with substance use carefully considered and managed to perform multiple simultaneous roles: relieving pain, intoxication, avoiding withdrawal symptoms, and managing other physical and mental conditions [28, 57, 58]. Given the obvious concerns associated with accessing the unregulated toxic drug supply during an overdose crisis, it is important that appropriate pain management be made available within healthcare settings to reduce the risks associated with PWUD self-managing their pain.

Our findings suggest that people receiving OAT may be more likely to be denied pain medication. These findings are especially concerning given the high prevalence of chronic pain among people receiving OAT, which a recent meta-synthesis estimated to be approximately 45% [59]. Concerns around moderate-to-severe acute pain management with opioids are also important as people maintained on opioids, including OAT, may have tolerance levels that requires an additional dosage to reach analgesia. There are many factors that may be contributing to the increased denial rate among this sub-sample of PWUD. Healthcare providers’ stigma and distrust towards PWUD regarding their pain may be magnified when patients have a formal opioid use disorder diagnosis. Further, physicians may be reluctant to prescribe additional medications for pain given that OAT can be optimized for pain management [60–63]. A previous investigation of these cohort studies found that 23% of participants who were enrolled in methadone maintenance therapy at the time of their request for prescription pain medication were told that they were denied because their methadone maintenance therapy was sufficient [48]. Physicians may also have concerns about co-prescribing other drugs, especially opioids, that may interfere with or not work alongside OAT. Finally, OAT clinics in this setting are often siloed and do not provide treatment deemed beyond the scope of OAT care, thus perpetuating a perceived dichotomization between pain treatment and substance use disorder treatment [26, 28, 48, 63, 64]. This is despite evidence that OAT can provide pain relief when carefully dispensed with concurrent treatment in mind, such as with different dosing schedules and amounts [60–63]. Given the high prevalence of chronic pain among PWUD engaged in OAT, future research should continue to investigate how best to deploy OAT, alongside other therapies, to manage pain while simultaneously supporting OAT treatment outcomes. For example, while there is strong evidence supporting the use of cannabinoids for pain relief [65], there is preliminary evidence that cannabis use might also support improved outcomes from OAT, including better retention and lower rates of exposure to the unregulated drug supply [66, 67].

Although there is some literature and province-specific practice recommendations on the topic [63, 68, 69], there are no clinical practice guidelines specific to the concurrent management of pain and substance use disorders in Canada, and none identified internationally [70]. Existing guidelines focus either on the treatment of pain or the treatment of substance use disorders, with only short sections dedicated to concurrent treatment consideration [21, 71–74]. The lack of clear, evidence-based guidelines can lead to some notable consequences. First, without guidelines for the management of pain among PWUD specifically, prescribers are left to individually adapt guidelines that are made on risk–benefit considerations that may not be relevant to PWUD. For example, the risk of dependence, overdose, and death from prescribed opioids may be considered differently for a patient with active unregulated opioid use who will rely on the unregulated supply for pain management if not supported in-clinic, as we see is common within our analysis. Second, unique considerations arise around contraindications between prescription pain medications and unregulated drugs or substance use disorder treatments. Such considerations include medications that affect the central nervous system (e.g., benzodiazepines, antipsychotics, anticonvulsants, and opioids including methadone) and can lead to potentially fatal respiratory depression [75]. Third, the lack of guidelines individualizes care to a greater degree, leaving PWUD seeking pain treatment to the preferences and competency level of their attending physician to a greater extent. As previously outlined, PWUD may face stigma, discrimination, and treatment refusal from healthcare providers when accessing treatment for pain, which can lead to consequences such as disengagement from care and self-management of pain [26, 28, 29, 76]. Our findings suggest that younger PWUD may experience greater barriers in accessing pharmaceutical pain management, evidenced by a 2% decrease in the odds of being denied medication per year older. This may be due to the types of acute and chronic pain more likely to be experienced by younger people, or it may be a result of the greater prevalence and subsequent normalization of chronic pain among older individuals. Further research is needed to understand this association. Fourth, the lack of guidelines specific to PWUD has left many healthcare providers hesitant and reticent when providing pain care to this complex patient population, restricted by a lack of confidence in what the best practices are [77]. This apprehension is further induced by physicians’ fear of potential repercussions from medical regulatory colleges if identified as overprescribing opioids [77, 78]. Future research among healthcare providers should investigate the role of psychosocial and structural factors in enabling or complicating the provision of analgesia. Finally, current guidelines suggest providing referrals to (or getting guidance from) addiction medicine specialists experienced in pain when treating patients with concurrent substance use disorder and acute or chronic pain [74]. This not only creates an additional step in accessing care which may act as a barrier to many PWUD, but also assumes an unrealistic capacity of addiction medicine specialists for conditions that could be treated in primary care if guidance was available [77]. Further, a recent review of multidisciplinary pain treatment facilities across Canada found that almost one in three centers actively exclude patients with a substance use disorder [79]. While guidelines highlight the importance of clinical judgement and shared decision making when deciding on therapeutic pathways [72], there is evidently a gap in clinical and patient resources for the management of pain amidst substance use.

We found that the denial rate of pain medications among PWUD was reasonably stable over time, with no significant change despite important contextual shifts including BC’s rapid increase in overdose deaths and new opioid prescribing guidelines in 2016 and 2017. In particular, the absence of a lasting increase in the opioid denial rate as of 2016 suggests that the guidelines may be mismatched with the realities of PWUD and their prescribers (e.g., minimal access to alternative non-pharmaceutical therapies, socio-economic marginalization, complex comorbidities) and are not sufficient to support the treatment of pain among PWUD [78]. Guidelines specific to the concurrent management of pain and substance use are needed to support those denied requested medications for pain and outline best practices among people receiving pain medications. Such guidelines may be especially helpful in treating patients who do not know what medications alleviate their pain. As seen in Fig. 1, participants whose pain medication requests were not specified at the time of data collection had the highest denial rate and would likely benefit from the existence of guidelines that would assist physicians in creating a treatment plan in collaboration with their patient. Further, given that the reported denial rate among participants who requested opioids was only 21%, it may be inferred that a substantial proportion received opioid prescriptions for pain despite the current restrictive guidelines that are based on evidence of limited comparative efficacy and serious harms associated with long-term use [18, 21, 72]. Revised guidelines will need to pay special attention to support best practices and informed clinical judgment regarding the appropriate use of opioids and other drugs at risk of misuse for PWUD experiencing pain. Opioid-specific considerations include how to discuss the risks and benefits of prescription opioids for pain with PWUD; approaches to the tapering or discontinuation of opioids; acute prescribing considerations for people with different types of substance use; how to minimize the effects of hyperalgesia; information on drug interactions and contraindications between opioids and other prescribed and unregulated drugs; and best standards around optimizing OAT for the concurrent management of pain and opioid use disorder. Finally, though beyond the scope of this analysis, research and guidance is also needed regarding non-pharmaceutical therapies to address the complexity of pain among PWUD.

This analysis has several limitations to consider. First, as this is an observational study, causation cannot be inferred. Second, there is potential for unmeasured confounding in our model. Potential confounders that could not be assessed include participants’ underlying health conditions, the clinical setting of the request (i.e., emergency doctor, primary doctor, community practitioner), and features of the pain for which a prescription was sought, such as the intensity or duration of the pain. Third, participants in the cohorts are not selected at random, with the community-based sampling methods limiting generalizability to broader populations of PWUD and other settings. Fourth, apart from HIV and HCV serostatus, our analysis relies on self-reported data which introduces the potential of recall and social desirability bias. However, research has shown self-reported data among PWUD to be reliable and valid [80–82]. Fifth, though we aimed to be inclusive of both chronic and acute pain experiences, our inclusion criteria of moderate or extreme pain or discomfort at the time of interview may have underestimated the participants who experienced acute pain during the follow-up period that resolved prior to their study visit, biasing the sample towards people with persistent pain. Further, we were not able to account for participants who may have requested pain medication multiple times within the study period, which may lead to a misestimation of the denial rate. Finally, in cases where requests included multiple types of medications, which medication may have triggered a denial could not be determined. To account for this, denial rates were further grouped by type of prescription requested (opioid, non-opioid, and non-specified requests).

## Conclusions

PWUD continue to report high rates of pain and, in our seven-year longitudinal study, a substantial proportion continue to be denied pain medication, with denial associated with various risks, including high-intensity heroin/unregulated opioid use, self-management of pain, and non-fatal overdose. Blanket recommendations against the use of opioids and other medications for pain management are insufficient and are mismatched with the realities of PWUD. Guidelines specific to the pharmaceutical management of pain among PWUD are needed to support the provision of appropriate and effective analgesia.

## Data Availability

Assurances of strict confidentiality given to participants during the consenting process preclude public sharing of datasets.

## Abbreviations

PWUD: People who use drugs
BC: British Columbia
VIDUS: The Vancouver injection drug users study
ACCESS: The AIDS care cohort to evaluate exposure to survival services
ARYS: The at-risk youth study
HIV: Human immunodeficiency virus
HCV: Hepatitis C virus
CAD: Canadian dollar
POC: Person of colour
GEE: Generalized estimating equations
IQR: Interquartile range
OR: Odds ratio
CI: Confidence interval
AOR: Adjusted odds ratio
OAT: Opioid agonist therapy
